# CILP-1 Is a Biomarker for Backward Failure and Right Ventricular Dysfunction in HFrEF

**DOI:** 10.3390/cells12242832

**Published:** 2023-12-13

**Authors:** Annika Weidenhammer, Suriya Prausmüller, Clemens Partsch, Georg Spinka, Bianca Luckerbauer, Mirella Larch, Henrike Arfsten, Ramy Abdel Mawgoud, Philipp E. Bartko, Georg Goliasch, Stefan Kastl, Christian Hengstenberg, Martin Hülsmann, Noemi Pavo

**Affiliations:** Department of Internal Medicine II, Clinical Division of Cardiology, Medical University of Vienna, 1090 Vienna, Austrianoemi.pavo@meduniwien.ac.at (N.P.)

**Keywords:** heart failure, CILP1, right heart, fibrosis

## Abstract

Background: CILP-1 regulates myocardial fibrotic response and remodeling and was reported to indicate right ventricular dysfunction (RVD) in pulmonary hypertension (PH) and heart failure (HF). This study examines CILP-1 as a potential biomarker for RVD and prognosis in heart failure with reduced ejection fraction (HFrEF) patients on guideline-directed medical therapy. Methods: CILP-1 levels were measured in 610 HFrEF patients from a prospective registry with biobanking (2016–2022). Correlations with echocardiographic and hemodynamic data and its association with RVD and prognosis were analyzed. Results: The median age was 62 years (Q1–Q3: 52–72), 77.7% of patients were male, and the median NT-proBNP was 1810 pg/mL (Q1–Q3: 712–3962). CILP-1 levels increased with HF severity, as indicated by NT-proBNP and NYHA class (*p* < 0.0001, for both). CILP-1 showed a weak–moderate direct association with increased left ventricular filling pressures and its sequalae, i.e., backward failure (LA diameter r_s_ = 0.15, *p* = 0.001; sPAP r_s_ = 0.28, *p* = 0.010; RVF r_s_ = 0.218, *p* < 0.0001), but not with cardiac index (CI) and systemic vascular resistance (SVR). CILP-1 trended as a risk factor for all-cause mortality (crude HR for 500 pg/mL increase: 1.03 (95%CI: 1.00–1.06), *p* = 0.053) but lost significance when it was adjusted for NT-proBNP (adj. HR: 1.00 (95%CI: 1.00–1.00), *p* = 0.770). No association with cardiovascular hospitalization was observed. Conclusions: CILP-1 correlates with HFrEF severity and may indicate an elevated risk for all-cause mortality, though it is not independent from NT-proBNP. Increased CILP-1 is associated with backward failure and RVD rather than forward failure. Whether CILP-1 release in this context is based on elevated pulmonary pressures or is specific to RVD needs to be further investigated.

## 1. Introduction

Heart failure with reduced ejection fraction (HFrEF) is characterized by a decreased left ventricular (LV) systolic function with a persistently increased LV preload [1,2]. Postcapillary pulmonary hypertension (PH) and right ventricular dysfunction (RVD) are long-term sequelae of chronic LV failure [3,4]. The development of RVD has been associated with a worse outcome and increased morbidity and mortality in patients with heart failure (HF) [3,5,6]. Still, research specifically addressing the impact of RVD in HFrEF is scarce. This is further complicated by the fact that the exact definition of RVD remains debatable, and there are currently no established diagnostic standards for RVD [1]. NT-proBNP, predominantly released by the LV upon myocardial stretch, is a biomarker with an excellent prognostic ability in HF [7]. However, NT-proBNP cannot entirely grasp HFrEF-related RVD [8]. A biomarker specifically indicating RV stress could further refine risk assessment in advanced HFrEF.

Right ventricular (RV) fibrosis is an important pathological feature of RVD [1]. Fibrillar collagen is the main component of the myocardial extracellular matrix, while the balance between extracellular matrix synthesis and degradation is determined by cardiac fibroblasts [9]. Transforming growth factor beta 1 promotes fibrosis by activating the signaling pathways that lead to profibrotic gene overexpression [10]. Cartilage intermediate layer protein 1 (CILP-1) is an extracellular matrix protein predominantly secreted from chondrocytes in the articular cartilage [11]; however, it is also expressed within the myocardium, where it is supposedly produced by cardiac fibroblasts [1,12]. CILP-1 plays a role in the counterregulatory mechanism of fibrosis and collagen remodeling, presumably by a paracrine mechanism, where it acts as an antagonist of the transforming growth factor beta 1 pathway and profibrotic gene expression [10,13,14]. Animal models have shown less progression of ventricular fibrosis and diastolic dysfunction under the overexpression of CILP-1 [10]. A study investigating a small cohort of HFrEF patients reported the presence of CILP-1 in the extracellular space of failing hearts, with a stronger signal in fibrous rich tissue [12]. Higher myocardial CILP-1 levels were also reported in patients with hypertrophic cardiomyopathy, and higher circulating CILP-1 levels were found in patients with dilated cardiomyopathy compared with controls [15].

To date, circulating CILP-1 has only been investigated in a small number of human studies with cardiac pathologies. CILP-1 is elevated in ischemic cardiomyopathy or PH [1,8]. In ischemic cardiomyopathy, increased CILP-1 was associated with adverse RV remodeling, while no association with LV parameters was observed. CILP-1 was also a good predictor of a right ventricular function (RVF) of <40%, suggesting that CILP-1 might be a new non-invasive biomarker for RVD [8]. A subsequent study examining chronic HF patients with a left ventricular ejection fraction (LVEF) of ≤50% reported that CILP-1 was a risk factor for increased mortality over a 1-year period [14]. Here, CILP-1 improved the risk prediction beyond NT-proBNP. These initial data suggest that it is worthwhile to further investigate CILP-1 in HFrEF.

This study aims to extend the evidence of circulating CILP-1 as a novel biomarker for RVD and prognosis in HFrEF patients by investigating CILP-1 levels in a large cohort of HFrEF on guideline-directed medical therapy (GDMT) and assessing its association with (i) cardiac morphology, function, hemodynamics; and (ii) outcome parameters such as unplanned HF hospitalizations and all-cause mortality.

## 2. Materials and Methods

The study was approved by the Institutional Review Board of the Medical University of Vienna (27 April 2023; Nr.: 1181/2022) and conducted in accordance with the current revision of the Declaration of Helsinki.

### 2.1. Patient Population

Consecutive patients with HFrEF were included from a prospective registry linked to biobanking at the outpatient HF clinic of the Medical University of Vienna between 2016 and 2022. Inclusion criteria for the registry were a diagnosis of stable HFrEF based on a documented LVEF < 40%, according to the current guidelines of the European society of cardiology [16]. Patients had to fulfill the criteria of being 18 years of age or older and had to provide written informed consent. Patients underwent routine clinical control visits and received standard of care. Demographic and clinical parameters, comorbidities, exact medications, and routine laboratory parameters were assessed at each visit. Parameters of right heart catheterization, whenever available, were documented. Patients were followed up for clinical events such as hospitalizations or death. For this study, patients were selected from the registry where biobank samples were available within 3 months of an echocardiographic examination, and in cases of multiple matches, the longest possible follow up was considered.

During follow up, all-cause mortality and hospitalizations due to cardiovascular reasons were documented. All-cause mortality was assessed by linking the registry to the Austrian Death Registry.

### 2.2. Echocardiography and Right Heart Cathetherization

Standard transthoracic echocardiographic assessments were performed by board-certified cardiologists using commercially available scanners (Vivid E9, GE Healthcare, Chicago, IL, USA) according to standardized protocols and current guidelines [17]. Cardiac morphology was assessed using diameters and volumes in four- and two-chamber views. A semiquantitative assessment of LV and RV function was performed by experienced readers using multiple acoustic windows and were presented as normal (EF > 50%), mildly reduced (EF 40–50%), moderately reduced (EF 30–40%), and severely reduced (EF < 30%) LV or RV function. Mitral (MR) and tricuspid regurgitation (TR) were quantified using an integrated approach as previously described. Systolic pulmonary artery pressure (sPAP) was calculated by adding the peak TR systolic gradient to the estimated central venous pressure. For a comprehensive evaluation of RV systolic function, the tricuspid annular plain systolic excursion (TAPSE), the RV lateral wall’s systolic movement using tissue Doppler imaging (RV-TDI), and the RV fractional area change (RV-FAC) were determined.

Right heart catheterization was available for a subgroup of patients. These patients underwent extended diagnostics and evaluation for special procedures, e.g., heart transplantation. Briefly, a 7F Swan-Ganz catheter (Baxter, Healthcare Corp, Munich, Germany) was inserted via femoral or jugular access and advanced via the superior vena cava, right atrium (RA), and RV to the pulmonary artery. CathCorLX (Siemens AG, Erlangen, Germany) was used to monitor pressures. The location of the catheter was determined by the waveform recorded by the tip of the catheter. At each site of interest, pressures were recorded by calculating the average throughout eight recorded cardiac cycles over a whole respiratory cycle. The RA and RV pressures and sPAP were recorded. After inflating the balloon and occluding the antegrade flow within the pulmonary artery, the pulmonary capillary wedge pressure (PCWP) was recorded. The cardiac output (CO) was measured by thermodilution. The transpulmonary gradient was derived by subtracting the PCWP from the mean sPAP. The pulmonary vascular resistance (PVR) was calculated by dividing the transpulmonary gradient by CO.

### 2.3. Determination of Circulating CILP-1 Levels

Serum CILP-1 concentrations were determined for the total cohort using a quantitative sandwich ELISA using the commercially available kit from Invitrogen (Human CILP-1 ELISA kit EH 109RBX5, Waltham, MA, USA), according to the manufacturer’s instructions and protocol. The assay has a detection range of 0.053–13 ng/mL, a minimum detectable concentration of 0.05 ng/mL, an intra-assay precision coefficient of variation of <10%, and an inter-assay precision coefficient of variation of <12%. For a subset of patients, CILP-1 levels were additionally measured using four kits: Antibodies-Online (CILP-1 ELISA kit, ABIN4882357), Ray Biotech (ELISA CILP), My BioSource (CILP-1 Elisa kit: MBS2887177), and Szabo Scandic/Cusabio (CSB-EL005437HU−96). Assays were performed according to the manufacturers’ instructions.

### 2.4. Statistical Analysis

Continuous data are expressed as median and interquartile range, while categorical variables are presented as percentages. Baseline characteristics are described in a descriptive way. The normality of the distribution of CILP-1 levels was tested using the Shapiro–Wilk test. To compare baseline parameters between CILP-1 tertiles, the Kruskal–Wallis test and Fisher’s exact test were used. To assess the association of CILP-1 levels with cardiac morphologic, functional, and hemodynamic data, the Spearman’s rho correlation coefficient was calculated or distributions were compared using the Kruskal–Wallis test. To estimate cardiac and hemodynamic predictors of CILP-1, a multiple linear regression analysis was performed by entering all available cardiac morphologic, functional, and hemodynamic data, consisting of the systemic vascular resistance (SVR), cardiac index (CI), left ventricular end-diastolic diameter (LVEDD), MR, left atrial (LA) diameter, PCWP, sPAP, PVR, RVF, right ventricular end-diastolic diameter (RVEDD), TR, and RA diameter. The association between CILP-1 and outcome measures such as all-cause mortality and hospitalizations due to cardiovascular reasons was analyzed by using a Cox proportional hazard regression analysis and Kaplan–Meier curves, with the Wilcoxon–Breslow–Gehan test being used for comparison between tertiles. To assess whether the association was independent of other factors, the model was adjusted for a clinical model (age, BMI, eGFR) and NT-proBNP. We applied a receiver operating characteristic (ROC) analysis to assess the predictive value of CILP-1 for RVD (reflected by RVEF < 40%, RV-FAC < 25%, TAPSE < 13 mm, and TAPSE/sPAP < 0.36 mm/mmHg) or outcome. CILP-1 cut-off values were determined based on the Youden Index. We have similarly repeated the analysis with NT-proBNP. The area under the curve (AUC) for the two biomarkers was compared using a software package in R Statistical Software (v4.1.2; R Core Team 2021, Vienna, Austria). Two-tailed analyses were used for all tests, and the significance level was set at *p* < 0.05. The statistical analysis was performed using SPSS^®^ software 26.0 for the Windows 10 OS.

## 3. Results

### 3.1. Baseline Characteristics of the Study Population

A total of 636 HFrEF patients were enrolled. CILP-1 values were not valid in 16 samples, leading to their exclusion from the analysis. The baseline characteristics of the total cohort, consisting of 610 patients, are presented in Table 1, the echocardiographic and hemodynamic characteristics in Table 2. The median age of the study cohort was 62 years (Q1–Q3: 52–72), with 475 patients (77.9%) being male, and the median NT-proBNP was 1810 pg/mL (Q1–Q3: 676–3978). A total of 71 patients (12.0%) were in New York Heart Association (NYHA) functional class I, 276 patients (46.7%) were in NYHA II, and 244 patients (41.3%) were in NYHA III/III+. An ischemic cause of HF was present in approximately half (55.6%) of the study population. Drug treatment for HF was well established, with 550 (90.2%), 561 (92.0%), 444 (72.8%), and 60 (9.8%) patients receiving medical therapy with renin angiotensin system inhibitors (RASi), beta-blockers (BB), mineralocorticoid receptor antagonists (MRA), and sodium-glucose co-transporter−2 inhibitors (SGLT2i), respectively. A majority of the patients achieved at least 50% of the target dose, with percentages of 53.4% (326), 70.5% (430), 69.8% (426), and 7.4% (45) for patients of the respective drug classes. Additionally, half (45.6%) of the study population received loop diuretics.

The median CILP-1 value was 3341 pg/mL (Q1–Q3: 2714–4170). In the tertile analysis, CILP-1 values ranged from 1354 to 2870 pg/mL for the low, from 2873 to 3813 pg/mL for the middle, and from 3820 to 33,480 pg/mL for the high tertile, with median values of 2520 pg/mL, 3341 pg/mL, and 4620 pg/mL, respectively. Higher CILP-1 values were observed for patients with more advanced HF, as reflected by higher NYHA class and NT-proBNP levels. Elevated CILP-1 levels were also found to be directly related to worsening renal or hepatic function, with increasing creatinine and urea and increasing gamma-glutamyl transferase, as well as decreasing butyrylcholinesterase in higher CILP-1 tertiles (*p* < 0.0001 for all).

### 3.2. Comparability of Different Commercially Available Human CILP-1 Assays

The comparison between the different commercially available immunoassay kits is shown in Supplementary Figure S1. CILP-1 levels assessed by Invitrogen correlated strongly with measurements by Antibodies-Online (r_s_ = 0.80; *p* < 0.0001) and RayBiotech (r_s_ = 0.80; *p* < 0.0001), as well as with Cusabio (r_s_ = 0.35, *p* = 0.001). However, there was no correlation with the results assessed by MyBiosource (r_s_ = 0.04; *p* = 0.71). 

### 3.3. CILP-1 Distribution and Association with HFrEF Severity

**Figure 1:**
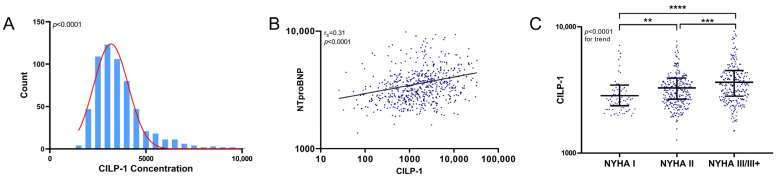
CILP-1 distribution and association with HF severity. (**A**) The distribution of CILP-1 is shown as a histogram. (**B**). The association of CILP-1 with NT-proBNP is shown as a scatterplot and as individual values indicating the median and interquartile range for different NYHA classes. For NT-proBNP, the Spearman correlation coefficient and level of significance is indicated. (**C**) For NYHA class, nonparametric tests (Mann–Whitney U and Kruskal–Wallis) were used for the comparisons between groups. *p* < 0.05 was considered significant. **—*p* ≤ 0.01; ***—*p* ≤ 0.001; ****—*p* ≤ 0.0001.

The distribution of CILP-1 and its association with HF severity are shown in Figure 1. CILP-1 revealed a non-normal distribution in HFrEF patients (*p* < 0.0001, Shapiro–Wilk test). CILP-1 showed a moderate correlation with NT-proBNP [r_s_ = 0.31, *p* < 0.0001] and increased with NYHA class (*p* < 0.0001 for trend). CILP-1 levels were higher for patients using loop diuretics (3608 pg/mL [2779–4415] vs. 3220 pg/mL [2645–3903], *p* = 0.002).

### 3.4. Association of CILP-1 with Echocardiographic and Hemodynamic Markers of Cardiac Dimensions and Function

**Figure 2:**
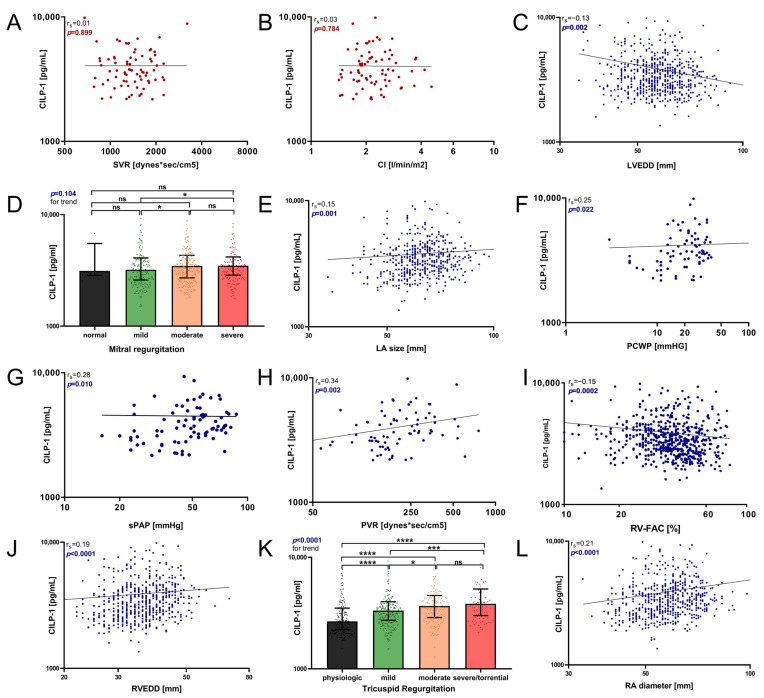
Association of circulating CILP-1 with cardiac function and hemodynamic parameter. The association of CILP-1 with (**A**) SVR, (**B**) CI, (**C**) LVEDD, (**D**) grade of MR, (**E**) LA size, (**F**) PCWP, (**G**) sPAP, (**H**) PVR, (**I**) RV-FAC, (**J**) RVEDD, (**K**) grade of TR, and (**L**) RA diameter is visualized as scatter and Tukey’s boxplots. Red represents forward failure, blue represents backwards failure. The Spearman correlation coefficients were calculated, and the Mann–Whitney U and Kruskal–Wallis non-parametrical tests were used for comparisons between groups. *p*-values are indicated within the respective plots. RA, right atrium; TR, tricuspid regurgitation; RV-FAC, right ventricular fractional area change; RV, right ventricle, sPAP, systolic pulmonary arterial pressure; PVR, pulmonary vascular resistance; PCWP, pulmonary capillary wedge pressure; LA, left atrium; MR, mitral regurgitation; LV, left ventricle; SVR, systemic vascular resistance; CI, cardiac index, n.s.—*p* > 0.05; *—*p* ≤ 0.05; ***—*p* ≤ 0.001; ****—*p* ≤ 0.0001.

Figure 2 summarizes the association between CILP-1 and cardiac morphologic and functional parameters as well as hemodynamic parameters. Increasing CILP-1 levels were associated with most markers of backward failure, such as increased LV load, i.e., LA size, mean PCWP, concomitant PH, i.e., sPAP and PVR, increased RV load, i.e., RV/RA size, grade of TR, and RV function mirrored by RV-FAC [LVEDD r_s_ = −0.13, *p* = 0.002; MR *p* = 0.104; LA diameter r_s_ = 0.15, *p* = 0.001; PCWP r_s_ = 0.25, *p* = 0.022; sPAP r_s_ = 0.28, *p* = 0.010; PVR r_s_ = 0.34, *p* = 0.002; RVEDD r_s_ = 0.19, *p* < 0.0001; RA diameter r_s_ = 0.21, *p* < 0.0001; TR *p* < 0.0001; RV-FAC r_s_ = −0.15, *p* = 0.002]. However, there was no association with parameters reflecting forward failure such as CI or SVR [*p* = 0.899; *p* = 0.784, respectively]. 

**Figure 3:**
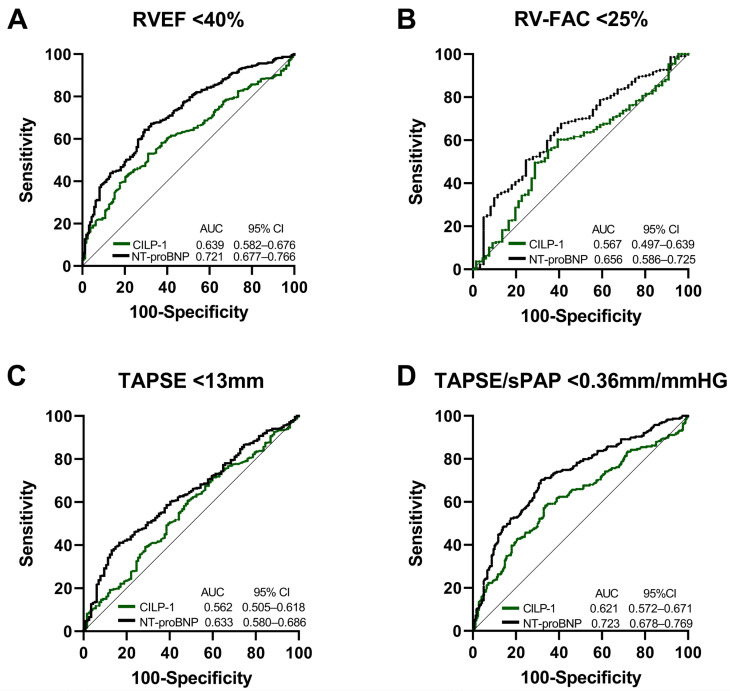
CILP-1 and NT-proBNP for the prediction of RVD in HFrEF. Receiver operating characteristic curve analysis for CILP-1 and NT-proBNP for predicting (**A**) RVEF < 40%, (**B**) RV-FAC < 25%, (**C**) TAPSE < 13 mm, and (**D**) TAPSE/sPAP < 0.36 mm/mmHG. The area under the curve and confidence intervals are indicated within the figures.

Figure 3 shows the ROC analysis of CILP-1 for the prediction of reduced RV function, i.e., RVEF < 40%, RV-FAC < 25%, TAPSE < 13 mm, and TAPSE/sPAP < 0.36 mm/mmHg, in comparison with NT-proBNP. The ability to predict RVD was comparably modest for both parameters indicated by AUCs below 0.7 for RV-FAC, TAPSE, and TAPSE/sPAP. For RVEF, CILP-1 showed an AUC of 0.64 with an optimal cut-off of 3272 ng/mL (sensitivity 69.93%, specificity 46.76%), whereas the AUC of NT-proBNP was higher at 0.72 and outperformed CILP-1 (*p* = 0.0038 for comparison).

In the multivariate model to predict high values of CILP-1, where all echocardiographic and hemodynamic features were entered into the model, the overall model had a poor performance (R^2^ = 0.391, *p* = 0.840). In a step-wise model, TR was the sole variable to enter the model (R^2^ = 0.067, Beta = 0.258, *p* = 0.039).

### 3.5. Association of CILP-1 with Prognosis in HFrEF

**Figure 4:**
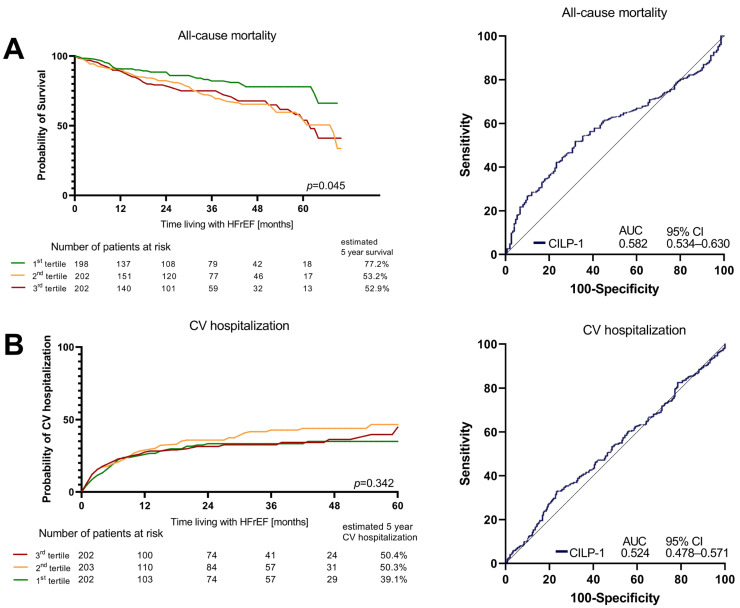
Survival and CV hospitalizations in HFrEF according to CILP-1 tertiles. Association of CILP-1 with (**A**) all-cause mortality and (**B**) Cardiovascular hospitalizations is shown as Kaplan–Meier plots for different CILP-1 tertiles and ROC analyses. For the Kaplan–Meier plots, the difference between the groups was assessed by the Wilcoxon–Breslow–Gehan test.

Over a median follow-up time of 26 months (Q1–Q3: 10–45), 156 (25.5%) patients from the total cohort died. Hospitalization due to cardiovascular reasons occurred in 247 (40.5%) patients. In the Cox regression analysis, CILP-1 levels showed a trend as a risk factor for all-cause mortality [crude HR for an increase of 500 pg/mL in CILP-1: 1.03 (95%CI: 1.00–1.06), *p* = 0.053]. After adjusting for the clinical model and NT-proBNP, CILP-1 was not significantly associated with mortality (adj. HR: 1.00, 95%CI: 1.00–1.00, *p* = 0.386 and adj. HR: 1.00, 95%CI: 1.00–1.00, *p* = 0.770). Better survival was only shown in the Kaplan–Meier analysis for the lowest CILP-1 tertile [*p* = 0.045, Wilcoxon–Breslow–Gehan]. Cardiovascular hospitalizations showed no association at all, neither in the Cox regression [crude HR for an increase of 500 pg/mL in CILP-1: 1.012 (95%CI: 0.98–1.05); *p* = 0.473] nor in the tertiles [*p* = 0.342, Wilcoxon–Breslow–Gehan], as shown in Figure 4.

## 4. Discussion

This study extends the evidence of circulating CILP-1 as a biomarker in HFrEF. Higher CILP-1 levels are associated with increasing HFrEF severity as well as functional impairment of the kidney and liver. Circulating CILP-1 was directly associated with parameters indicating increased LV filling pressures and its sequelae, i.e., LA size, mean PCWP, PVR, sPAP, grade of TR, and RV-FAC, but not with CO or SVR. CILP-1 showed limited performance in predicting RVD with an RVEF of <40%. CILP-1 showed a trend as a risk marker for mortality and loses its impact upon adjustment to NT-proBNP. Cardiovascular hospitalization is not associated with CILP-1 levels.

### 4.1. Comparison of CILP-1 Levels in CV Disease and HF

To the best of our knowledge, these are the first data directly correlating commercially available CILP-1 assays in HFrEF. Our results demonstrate an excellent correlation between the assays from Invitrogen, Antibodies-Online and RayBiotech and a good correlation with Cusabio. However, the assay from MyBioSource showed non-comparable results. Unlike other commercially available kits that measure N-terminal CILP-1, the antibody in the MyBiosource assay targets the cleavage site of the precursor protein proCILP-1, measuring full-length CILP-1 [15]. This could lead to essentially different results.

Until now, only three manuscripts have reported circulating CILP-1 levels in healthy individuals [1,8,13]. They used the commercially available kits from MyBiosource and Cusabio [1,8]. In the first study using the MyBiosource kit, CILP-1 values were around 2 ng/mL in healthy individuals, while levels in HF patients were lower [13]. This is in contrast with other previous findings and was attributed to the different analytic properties of the assay. In the other two studies [1,8] conducted by the same group using the Cusabio assay, healthy individuals showed 1389 pg/mL (Q1: 860, Q3: 2214) and 2913 pg/mL (Q1: 2436, Q3: 3293), respectively, while CILP-1 values for HF patients were higher at 3431 pg/mL (Q1: 2383, Q3:4980) and 4164 pg/mL (Q1: 2926, Q3: 5429), respectively [1,8]. Another paper used the Invitrogen assay in HFrEF patients and reported a median CILP-1 level of 3.92 ng/mL (Q1–Q3 2.62–6.12) [14]. These values are consistent with our results. 

### 4.2. Association of CILP-1 with Disease Severity and Functional and Hemodynamic Parameters in HF and Outcome

Our data show a clear direct association of CILP-1 levels with HF severity, as reflected by NYHA and NT-proBNP levels. This finding aligns with other reports in human cardiovascular disease [8]. Two recent studies suggested that CILP-1 might also be a useful biomarker in particular for RVD [1,8]. In these manuscripts, patients with PH had higher CILP-1 levels compared with patients with dilated cardiomyopathy and preserved RV function, while maladaptive RVD in PH was characterized by even higher levels [1]. Maladaptive RVD was defined by reduced RV function, as indicated by TAPSE, CI, RV dilation, and increased mean PAP [1]. However, in contrast to patients with other forms of PH, CI and PAP are not surrogates for RVF. These results are not entirely comparable to ours. In previous reports, the ROC analysis showed that NT-proBNP and CILP-1 were good and comparable predictors of maladaptive RVD, with the ideal CILP-1 cut-off value being calculated at 4373 pg/mL [1]. A small cohort of 98 patients with iCMP assessed cardiac function by magnetic resonance imaging and reported that low RVEF and RV dilation were associated with increasing CILP-1 levels but LV parameters were not [8]. In the ROC analysis, circulating CILP-1 was a good predictor of RVD, i.e., RVEF < 40%, but not LV dysfunction [8]. The cut-off value was calculated at 3545 pg/mL. In a multivariable regression including RVEF, NT-proBNP, the presence of atrial fibrillation, and GFR, only RVEF remained a significant predictor of CILP-1 levels [8]. Our data, representing the first data from a relatively large and unselected HFrEF patient cohort, confirm these previous findings in part.

CILP-1 levels increased with worsening RV function and indicators of remodeling, but also with signs of elevated LV filling pressure, pulmonary pressure, and grade of TR. However, no such association was observed with MR. The pathophysiology of MR is still poorly understood [18]. Milton Packer argues that the nature of secondary MR is multifaceted, comprising both proportionate forms, where the degree of MR is proportionate to the degree of LV dilatation; and disproportionate forms, where the degree of MR is higher than as predicted by the LV end-diastolic volume [19]. This diversity potentially explains why MR exhibits no direct correlation with CILP-1, unlike other biomarkers that are mainly indicative of LV dilatation [20,21]. This might be the reason why MR is not directly related to CILP-1, unlike other markers of LV function.

In contrast to that, and consistent with previous findings, no correlation was observed between CILP-1 and forward function, such as CO. This aligns with the hypothesis that CILP-1 is instead a biomarker for backward failure and RVD, even though the association of CILP-1 with none of these parameters was highly pronounced. Our data also showed a rather limited ability of CILP-1 for the prediction of RVEF < 40%, which was outperformed by NT-proBNP.

The multilinear regression model incorporating all echocardiographic and hemodynamic data yielded a low R^2^ value, suggesting that the main predictors of CILP-1 lie beyond obvious cardiac features.

Following previous studies on the association of CILP-1 and RVD, data from 210 patients with chronic HF, including patients with LVEF < 50%, were evaluated to assess the ability of CILP-1 to predict the 1-year survival [14]. CILP-1 was significantly associated with all-cause mortality in the crude analysis, with a 1.61-fold increase in mortality per standard deviations, and the association remained significant after adjustment for NT-proBNP and in a model that also included age and renal function [14]. In contrast, the present study, which included 636 patients with a median follow-up period of over 2 years and recorded 156 events, only demonstrated a weak association between CILP-1 and survival. In the Cox regression analysis, CILP-1 showed a trend toward being a risk factor for all-cause mortality, although this association was not independent of NT-proBNP or clinical factors (age, BMI, eGFR). Additionally, increased CILP-1 levels were not associated with cardiovascular hospitalizations.

## 5. Conclusions

This study represents the largest investigation of CILP-1 in HFrEF on GDMT up to this point. Circulating CILP-1 levels demonstrate a direct correlation with HF severity. Elevation in CILP-1 levels seem to reflect backward failure, accompanying increased LV filling pressures, pulmonary arterial pressures, PVR, deterioration in right heart dimensions, and RVF, but not CI. Nevertheless, CILP-1 shows a limited ability in predicting RVD and all-cause mortality overall.

### Limitations

This study is a single-center study. The gold standard for functional RV assessment is RVEF, derived from cardiac magnetic resonance imaging (cMRI). In this paper, we have used a 2D echocardiography-derived parameter of RV systolic function in the form of TAPSE, RV-TDI, and RV-FAC, which was shown to correlate well with cMR.I [22].

## Figures and Tables

**Table 1:** Baseline characteristics of the total HFrEF patient cohort and comparison of parameters according to circulating CILP-1 tertiles. Continuous variables are given as median and 25th and 75th percentile; counts are given as numbers and percentages. Comparison between tertiles of CILP-1 were performed by the Kruskal–Wallis test and Fisher’s exact test; the *p*-value is indicated.

	Total Cohort (*n* = 610)	CILP-1 Tertile 1 (*n* = 203)	CILP-1 Tertile 2 (*n* = 204)	CILP-1 Tertile 3 (*n* = 203)	*p*-Value
CILP-1, pg/mL (Q1–Q3) Range	3341	2520	3341	4620	-
	(2714–4170)	(2249–2715)	(3116—3615)	(4169–5951)	
	1354–33,480	1354–2870	2873–3813	3820–33,480	
Basic demographics					
Age, years (Q1–Q3)	62 (52–72)	57 (46–67)	66 (56–73)	65 (55–75)	**<0.0001**
Male gender, *n* (%)	475 (77.9%)	150 (73.9)	170 (83.3)	155 (76.4)	0.058
BMI, kg/m^2^ (Q1–Q3)	27.7 (23.9–31.0)	27.7 (24.2–31.6)	28.3 (24.7–31.1)	26.4 (22.9–30.1)	**0.009**
Systolic BP, mmHg (Q1–Q3)	125 (110–140)	120 (110–135)	125 (110–142)	129 (110–140)	0.154
Diastolic BP, mmHG (Q1–Q3)	78 (70–85)	77 (70–85)	80 (70–85)	75 (70–87)	0.826
Heart rate, bpm (Q1–Q3)	70 (61–81)	70 (60–79)	69 (61–80)	70 (62–84)	0.668
NYHA class, *n* = 591; *n* (%)					**<0.0001**
I	71 (12.0%)	39 (19.9%)	22 (11.3%)	10 (5.0%)	
II	276 (46.7%)	93 (47.4%)	101 (51.8%)	82 (41.0%)	
III/IV	244 (41.3%%)	64 (32.7%)	72 (36.9%)	108 (54.0%)	
Comorbidities					
Coronary artery disease, *n* (%)	339 (55.6%)	109 (53.7%)	109 (53.4%)	121 (59.6%)	0.373
Atrial fibrillation, *n* (%)	131 (21.5%)	29 (14.3%)	46 (22.5%)	56 (27.6%)	**0.004**
Diabetes mellitus, *n* (%)	221 (36.2%)	56 (27.6%)	75 (36.8%)	90 (44.3%)	**0.014**
Arterial hypertension, *n* (%)	328 (53.8%)	100 (49.3%)	111 (54.4)	117 (57.6%)	0.453
Chronic kidney disease, *n* = 601; *n* (%)	319 (53.1%)	80 (40.0%)	114 (56.7%)	125 (62.5%)	**<0.0001**
Dyslipidemia *n* = 597; *n* (%)	227 (38.0%)	95 (47.7%)	69 (34.5%)	63 (31.8%)	0.002
Medication and device therapy					
Beta-blocker, *n* (%)	561 (92.0%)	186 (91.6%)	188 (92.2%)	187 (92.1%)	0.633
ACEi, *n* (%)	275 (45.1%)	94 (46.3%)	99 (48.5)	82 (40.4%)	0.338
ARB, *n* (%)	109 (17.9%)	29 (14.3%)	35 (17.2%)	45 (22.2%)	0.239
ARNI, *n* (%)	166 (27.2%)	54 (26.6%)	54 (26.5%)	58 (28.6%)	0.612
MRA, *n* (%)	444 (72.8%)	150 (73.9%)	157 (77.0%)	137 (67.5%)	0.203
SGLT2 inhibitors, *n* (%)	60 (9.8%)	27 (13.3%)	16 (7.8%%)	17 (8.4%)	0.263
Ivabradin, *n* (%)	37 (6.1%)	12 (5.9%)	9 (4.4%)	16 (7.9%)	0.401
Diuretics, *n* (%)	278 (45.6%)	83 (40.9%)	84 (41.2%)	111 (54.7%)	**0.006**
ICD, *n* (%)	251 (41.1%)	85 (41.9%)	81 (39.7%)	85 (41.9%)	0.391
CRT, *n* (%)	163 (26.7%)	42 (20.7%)	64 (31.4%)	57 (28.1%)	**0.025**
PM, *n* (%)	58 (9.5%)	21 (10.3%)	15 (7.4%)	22 (10.8%)	0.316
Laboratory parameters					
NT-proBNP, pg/mL (Q1–Q3)	1810.0 (676.1–3978.0)	923.7 (412.9–2232.8)	1907.0 (882.9–4138.5)	2656.0 (1326.3–5928.5)	**<0.0001**
eGFR, mL/min/1.73 m²	57.7 (41.9–75.3)	65.2 (49.5–80.2)	56.9 (41.2–70.6)	49.7 (37.6–68.1)	**<0.0001**
CREA, mg/dL (Q1–Q3)	1.21 (0.98–1.58)	1.11 (0.90–1.37)	1.25 (1.01–1.58)	1.35 (1.06–1.76)	**<0.0001**
Urea, mmol/L (Q1–Q3)	23.7 (17.1–33.6)	20.9 (15.9–28.9)	24.1 (17.5–34.4)	26.6 (17.1–36.7)	**<0.0001**
Sodium, mmol/L (Q1–Q3)	140.0 (138.0–141.0)	140.0 (137.3–141.8)	140.0 (138.0–141.0)	140.0 (138.0–141.0)	0.876
Potassium, mmol/L (Q1–Q3)	4.8 (4.4–5.1)	4.8 (4.4–5.1)	4.8 (4.4–5.1)	4.7 (4.4–5.1)	0.696
Total bilirubin, mg/dL (Q1–Q3)	0.6 (0.4–0.8)	0.5 (0.4–0.8)	0.6 (0.4–0.8)	0.6 (0.4–0.9)	0.064
BChE, U/L (Q1–Q3)	7.01 (5.67–8.36)	7.77 (6.47–8.76)	6.91 (5.67–8.09)	6.50 (5.19–7.78)	**<0.0001**
AST (GOT), U/L (Q1–Q3)	24 (19–29)	23 (19–27)	24 (20–29)	25 (19–31)	0.070
ALT (GPT), U/L (Q1–Q3)	23 (17–33)	23 (17–32)	22 (16–34)	23 (16–33)	0.714
GGT, U/L (Q1–Q3)	44 (26–91)	36 (21–61)	43 (24–94)	63 (32–124)	**<0.0001**
Total cholesterol, mg/dL (Q1–Q3)	154 (124–188)	163 (131–196)	151 (121–185)	149 (124–188)	**0.035**
Hemoglobin, g/dL (Q1–Q3)	13.6 (12.2–14.7)	13.7 (12.6–14.8)	13.7 (12.2–14.7)	13.4 (11.8–14.7)	0.062
Ferritin, µg/L (Q1–Q3)	150.8 (81.4–256.2)	153.4 (65.2–260.2)	147.4 (68.3–256.3)	155.3 (94.1–251.3)	0.553
Transferrin saturation, % (Q1–Q3)	21.4 (14.7–30.0)	24.3 (16.6–31.3)	20.6 (14.2–29.6)	19.9 (14.3–28.7)	**0.027**
Uric acid, µmol/L (Q1–Q3)	6.8 (5.5–8.2)	6.6 (5.3–7.8)	6.9 (5.7–8.2)	7.0 (5.8–8.9)	**0.009**
Leukocyte count, G/L (Q1–Q3)	7.6 (6.3–8.9)	7.8 (6.6–9.0)	7.4 (6.1–8.9)	7.6 (6.3–9.1)	0.428
CRP, mg/dL (Q1–Q3)	0.3 (0.1–0.7)	0.3 (0.1–0.6)	0.3 (0.1–0.6)	0.4 (0.2–1.1)	**0.002**

CILP-1—Cartilage intermediate layer protein 1; BMI—body mass index; BP—blood pressure; NYHA—New York Heart Association; ACEi—angiotensin-converting enzyme inhibitors; ARB—angiotensin receptor blockers; ARNI—angiotensin receptor-neprilysin inhibitors; MRA mineralocorticoid receptor blockers; SGLT2—sodium-dependent glucose co-transporter 2; ICD—implantable cardioverter defibrillator; CRT—cardiac resynchronization therapy; PM—pacemaker; NT-proBNP—N-terminal pro-brain natriuretic peptide; CREA—creatinine; eGFR—estimated glomerular filtration rate; BChe—butyrylcholinesterase; AST—aspartate aminotransferase; ALT—alanin aminotransferase; GGT—gamma-glutamyl transferase; CRP—C-reactive protein. n = 610 patients; if data are not complete, an individual n is given.

**Table 2:** Echocardiographic and hemodynamic characteristics of the HFrEF patient cohort. Continuous variables are given as the median and 25th and 75th percentile; counts are given as numbers and percentages.

Echocardiographic Parameters	Total Cohort *n* = 610	CILP-1 Tertile 1 (*n* = 203)	CILP-1 Tertile 2 (*n* = 204)	CILP-1 Tertile 3 (*n* = 203)	*p*-Value
LVEF, *n* (%) recovered/mildly/ moderately/severely reduced	22 (3.6%), 35 (5.7%). 87 (14.3%), 466 (76.4%)	11 (5.4%), 12 (5.9%), 31 (15.3%), 149 (73.4%)	7 (3.4%), 12 (5.9%), 27 (13.2%), 158 (77.5%)	4 (2.0%), 11 (5.4%), 29 (14.3%), 159 (78.3%)	0.670
LVEDD, mm (Q1–Q3)	57 (51–64)	58 (51–65)	58 (52–64)	55 (49–61)	**0.003**
LA Diameter, mm (Q1–Q3)	62 (56–70)	58 (54–66)	64 (58–71)	63 (58–69)	**<0.0001**
RVEF, reduced, *n* (%) normal/mildly/moderately/severely reduced	252 (41.3%), 144 (23.6%), 135 (22.1%), 79 (13.0%)	113 (55.7%), 48 (23.6%), 26 (12.8%), 16 (7.9%)	70 (34.3%), 53 (26.0%), 50 (24.5%), 31 (15.2%)	69 (34.0%), 43 (21.2%), 59 (29.1%), 32 (15.8%)	**<0.0001**
TDI-RV m/s (Q1–Q3)	0.10 (0.08–0.12)	0.11 (0.09–0.13)	0.09 (0.08–0.12)	0.10 (0.08–0.12)	**0.002**
RV-FAC, % (Q1–Q3)	39 (30–48)	43 (34–51)	37 (29–47)	37 (28–48)	**<0.0001**
TAPSE, mm (Q1–Q3)	17 (13–20)	18 (14–21)	16 (13–20)	16 (12–19)	**<0.0001**
RVEDD, mm (Q1–Q3)	36 (31–40)	34 (30–38)	37 (32–41)	37 (33–42)	**<0.0001**
RA diameter, mm (Q1–Q3)	58 (51–65)	54 (48–61)	59 (52–68)	60 (54–65)	**<0.0001**
MR, n = 599; *n* (%) no/mild/moderate/severe	17 (2.8%), 202 (33.7%), 237 (39.6%), 143 (23.9%)	7 (3.5%), 84 (42.0%), 72 (36.0%), 37 (18.5%)	5 (2.5%), 57 (28.5%), 80 (40.0%), 58 (29.0%)	5 (2.5%), 61 (30.7%), 85 (42.7%), 48 (24.1%)	0.051
TR, n= 606; *n* (%) physiologic/mild/moderate/severe	155 (25.6%), 158 (26.1%), 180 (29.7%), 113 (18.6)	72 (36.0%), 55 (27.5%), 50 (25.0%), 23 (11.5%)	42 (20.6%), 61 (29.9%), 66 (32.4%), 35 (17.2%)	41 (20.3%), 42 (20.8%), 64 (31.7%), 55 (27.2%)	**<0.0001**
sPAP, mmHg (Q1–Q3)	44 (35–56)	38 (30–51)	48 (39–58)	46 (37–60)	**<0.0001**
**Hemodynamic parameters (*n* = 84)**	**Total cohort** **(*n* = 84)**	**CILP-1 Tertile 1** **(*n* = 16)**	**CILP-1 Tertile 2** **(*n* = 32)**	**CILP-1 Tertile 3** **(*n* = 36)**	
RA pressure mean, mmHg (Q1–Q3)	11 (7–18)	9 (4–15)	11 (5–18)	15 (9–18)	0.063
RV pressure end-diastolic, mmHg (Q1–Q3)	11 (8–16)	9 (6–17)	11 (8–16)	12 (9–18)	**0.023**
Mean PA pressure, mmHg (Q1–Q3)	33 (24–44)	25 (19–32)	35 (21–46)	36 (29–44)	**0.021**
PCWP, mmHg (Q1–Q3)	22 (14–29)	15 (10–23)	23 (13–31)	24 (17–29)	**0.021**
AV O2, %/mL (Q1–Q3)	68.7 (56.2–81.4)	65.2 (50.6–79.1)	72.4 (60.0–91.2)	66.8 (56.3–78.6)	0.187
RV max, mmHg/sec (Q1–Q3)	317 (248–410)	274 (213–361)	321 (237–414)	331 (256–424)	0.348
Stroke volume, mL/stroke (Q1–Q3)	55.4 (45.9–70.7)	62.4 (49.0–89.9)	55.6 (43.3–70.6)	55.1 (46.2–67.4)	0.294
Cardiac index/L/min/m^2^ (Q1–Q3)	2.1 (1.8–2.6)	2.3 (1.8–3.0)	2.1 (1.8–2.6)	2.1 (1.9–2.5)	0.596
PVR, dynes × sec/cm^5^ (Q1–Q3)	189 (150–280)	168 (146–184)	188 (131–296)	239 (176–324)	0.064
SVR, dynes × sec/cm^5^ (Q1–Q3)	1358 (1100–1721)	1331 (1067–1600)	1454 (1236–1728)	1334 (1065–1843)	0.740
TPR, dynes × sec/cm^5^ (Q1–Q3)	634 (454–853)	408 (302–553)	648 (430–855)	660 (599–815)	**0.009**
TSR, dynes × sec/cm^5^ (Q1–Q3)	1570 (1336–2009)	1489 (1163–1884)	1683 (1326–2021)	1530 (1336–2048)	0.544

LVEF—Left ventricular ejection fraction; LVEDD—left ventricular end-diastolic dimension; LA—left atrium; RVEF—right ventricular ejection fraction; TDI—tissue Doppler imaging right ventricle; RV-FAC—right ventricle fractional area change; TAPSE—tricuspid annular plane systolic excursion; RVEDD—right ventricular end-diastolic dimension; RA—right atrium; MR—mitral regurgitation; TR—tricuspid regurgitation; sPAP—systolic pulmonary arterial pressure; RA—right atrium; RV—right ventricle; PA—pulmonal artery; PCWP—pulmonary capillary wedge pressure; AV—atrioventricular; RV—right ventricle; PVR—pulmonary vascular resistance; SVR—systemic vascular resistance; TPR—total peripheral resistance; TSR—total systemic resistance. n = 610 patients; if data are not complete, an individual *n* is given.

## Data Availability

Data are available on request. The data presented in this study are available on request from the corresponding author. The data are not publicly available due to ethical restrictions.

## Supplementary Materials

The following supporting information can be downloaded at: https://www.mdpi.com/article/10.3390/cells12242832/s1, Figure S1: Comparison between different commercially available CILP-1 immunoassay kits.

## Abbreviations

AUC	Area under the curve
BB	Beta-blocker
CI	Cardiac index
CILP-1	Cartilage intermediate layer protein 1
cMRI	Cardiac magnet resonance imaging
CO	Cardiac output
GDMT	Guideline-directed medical therapy
HF	Heart failure
HFrEF	Heart failure with reduced ejection fraction
LV	Left ventricle
LVEF	Left ventricular ejection fraction
LVEDD	Left ventricular end-diastolic diameter
MR	Mitral regurgitation
MRA	Mineralocorticoid-receptor antagonist
NYHA	New York Heart Association
PCWP	Pulmonary capillary wedge pressure
PH	Pulmonary hypertension
PVR	Pulmonary vascular resistance
RASi	Renin–angiotensin system inhibitor
RA	Right atrium
RV	Right ventricle
RVD	Right ventricular dysfunction
RVEF	Right ventricular ejection fraction
RVEDD	Right ventricular end-diastolic diameter
RVF	Right ventricular function
RV-FAC	Right ventricular fractional area change
ROC	Receiver operating characteristic
SGLT2i	Sodium-glucose transporter 2 inhibitor
sPAP	Systolic pulmonary artery pressure
SVR	Systemic vascular resistance
TAPSE	Tricuspid annular plane systolic excursion
TR	Tricuspid regurgitation
