# Supplementary material for: CILP-1 Is a Biomarker for Backward Failure and Right Ventricular Dysfunction in HFrEF
Source: Cells. 2023 Dec 13;12(24):2832. doi: 10.3390/cells12242832 (PMC10741695; doi:10.3390/cells12242832)
Supplement: Supplementary file 1 [file cells-12-02832-s001.zip › cells-2717517-supplementary.pdf]

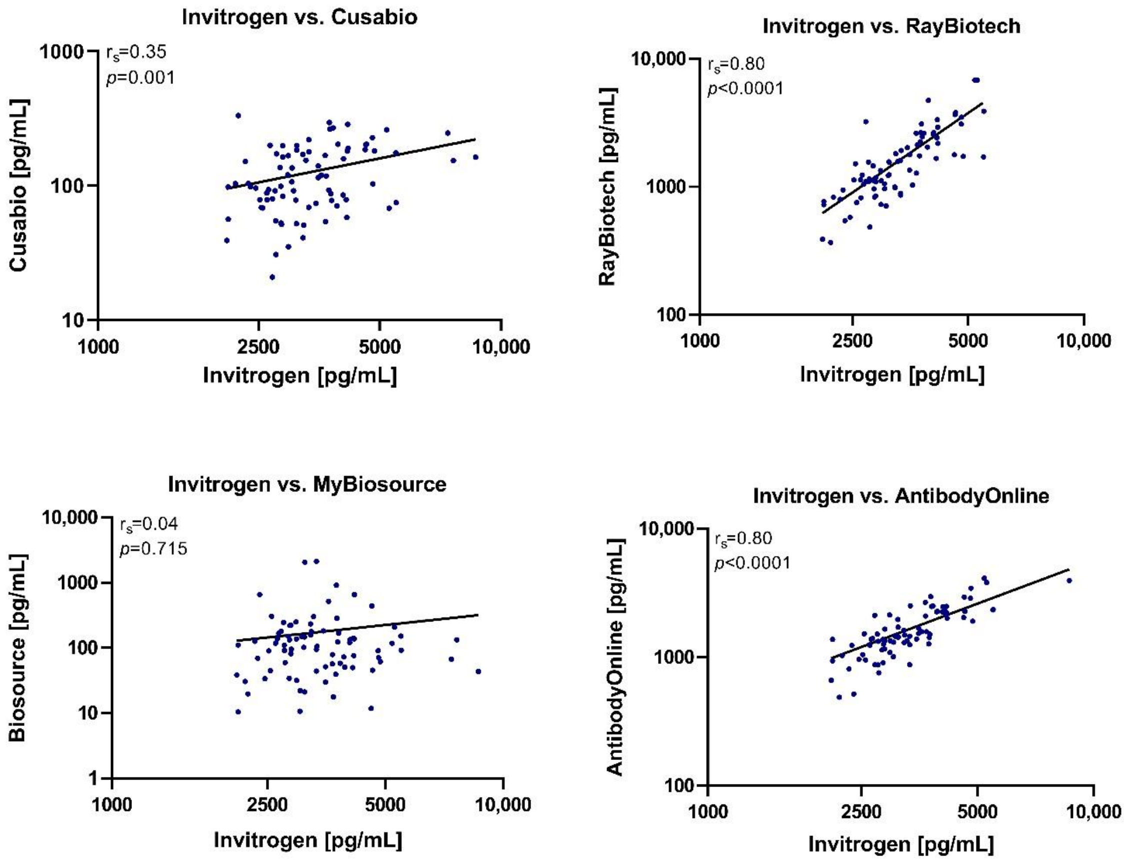

Supplementary Figure S1. Comparison between different commercially available CILP-1 immunoassay kits. The association of CILP-1 between ELISA kits is visualized as scatter plot, the p values are indicated within the respective plots. The Spearman correlation coefficients were calculated.
